# Impact of Nanoparticles Additions on the Strength of Dental Composite Resin

**DOI:** 10.1155/2022/1165431

**Published:** 2022-07-05

**Authors:** Emad Azmy, Mohamed Reda Zaki Al-Kholy, Mohamed Fattouh, Laila Mohamed Mohamed Kenawi, Mohamed Ahmed Helal

**Affiliations:** ^1^Elmarg Students' Clinic, General Authority of Health Insurance, Western Elmarg Area, Cairo, Egypt; ^2^Department of Removable Prosthodontics, Faculty of Dental Medicine, Al-Azhar University, Cairo, Egypt; ^3^Department of Fixed Prosthodontics, Faculty of Dentistry, Cairo University, Cairo, Egypt; ^4^Department of Oral and Maxillofacial Surgery, Faculty of Dentistry, Umm Al Qura University, Makkah, Saudi Arabia; ^5^Department of Endodontics, Faculty of Dentistry, Cairo University, Cairo, Egypt; ^6^Conservative and Restorative Dentistry Department, Faculty of Dentistry, Umm Al Qura University, Makkah, Saudi Arabia

## Abstract

**Objective:**

This study aimed to evaluate the effect of nanoparticles, zirconium dioxide (ZrO_2_), titanium dioxide (TiO_2_), and silicon dioxide (SiO_2_), on flexural strength (FS), hardness, and wear resistance of light cured dental composite resin.

**Materials and Methods:**

210 rectangular and disc-shaped composite resin specimens were fabricated with dimensions (25 × 2 × 2 ± 0.03 mm) and (6×4 ± 0.03 mm) for FS, hardness, and wear resistance, respectively (70/test). Specimens of each test were divided according to nanofillers into four groups, unmodified as control, ZrO_2_ (Z), TiO_2_ (T), and SiO_2_ (S) groups; each one was further subdivided into two subgroups according to nanoparticles concentration, 3wt.% and 7wt.% (Z3, Z7, T3, T7, S3, and S7), 10 specimens of each subgroup. A3-point bending test and Vickers hardness test were used for FS and hardness measurements, respectively. Wear resistance was evaluated by the differences in surface roughness of tested specimens before and after wear test. Two-way and 1-way ANOVA and Bonferroni's post hoc tests were done for data analysis (*α* = 0.05).

**Results:**

Two-way ANOVA for FS and wear resistance showed that there was a significant interaction between type of nanoparticles and concentration of nanoparticles (*p* < 0.001) while two-way ANOVA for hardness showed that both type of nanoparticles and concentration of nanoparticles had a significant effect (*p* < 0.001), while the effect of their interaction was not statistically significant (*p*=0.142). 1-way-ANOVA test showed significant increase in FS and wear resistance for all tested groups (*p* < 0.001 and *p* < 0.001, respectively) except T7 and S7. Also, there was a significant enhancement in hardness for all tested groups (*p* < 0.001).

**Conclusion:**

Modification of light cured composite resin with certain amounts of nanoparticles (3% and 7% of ZrO_2_ and 3% of TiO_2_ and SiO_2_) can be beneficial in improving flexural strength and wear resistance while hardness of composite resin was increased with all NPs additions.

## 1. Introduction

The introduction of dental resin-based composites (DRCs) in the last century was one of the most important steps in dentistry; since its introduction, it has undergone tremendous improvements enabling them to be more popular over dental amalgams in posterior and anterior teeth [1]. Good esthetics, bonding to tooth structure, and moderate cost compared with ceramics and conservative tooth preparation are the main advantages of DRCs.

DRCs have been used in many applications concerning prosthodontics as recontouring of buccal surface of abutments to provide undercuts for retainers of removable partial denture (RPD), occlusal or cingulum rest seat to support RPD [2, 3], reestablishment of patient's vertical dimension as well as construction of artificial denture teeth [4,5]. On the other hand, polymerization shrinkage, low wear resistance, and water sorption make it difficult to achieve a good restoration for long time [6].

Recently, nanotechnology has been used in dentistry in many fields, especially material improvement purposes. Reinforcement of DRCs by metal oxide nanoparticles is crucial for improvement of their mechanical properties as wear resistance, flexural strength, tensile strength, and fracture toughness leading to enhanced durability of the restoration [7,8].

Nanoparticles (0.1–100 nm) are characterized by their small size and large specific surface area which leads to their unique properties as good mechanical, chemical, optical, and magnetic properties when compared to their bulk ones [9]. Moreover, nanoparticles have a strong tendency to aggregate that may decrease the chemical interaction between them and organic matrix, so treatment of inorganic filler with silane coupling agent can improve the bond strength between the nanofiller and the resin, subsequently enhancing its properties [10].

Among commonly used nanoparticles are silicon dioxide (SiO_2_), titanium dioxide (TiO_2_), and zirconium dioxide (ZrO_2_). ZrO_2_ nanoparticles are ceramic materials that receive attention because of their unique properties as high strength, low abrasion, biocompatibility, esthetic acceptability, and desirable optical properties [11]. Hameed and Abdul Rahman reported that flexural strength of composite resin was significantly increased by adding ZrO_2_ [12].

TiO_2_ nanoparticles gained their importance due to their biocompatibility, corrosion resistant, high microhardness, chemical stability with high strength, and antimicrobial activities as well as their availability and low cost. It has been used as additive filler to composite resin enhancing its mechanical, physical, and optical properties; many studies reported that DRCs reinforced with treated TiO_2_ nanoparticles exhibited significantly higher wear resistance, flexural strength, and surface hardness [10,13].

SiO_2_ nanoparticles have good electrical insulation and thermal stability as well as high abrasion resistance. Azmy et al. found that addition of SiO_2_ nanoparticles to acrylic resin denture bases would increase color stability after being immersed in beverage solutions [14]. Tian et al. found that incorporation of SiO_2_ particles to composite resin significantly improved the flexural strength [15]. In another study, reinforcement of DRC with SiO_2_ led to significant increase in hardness [16]. However, Helal et al. recommended incorporating SiO_2_ particles cautiously in PMMA denture teeth as they may decrease wear resistance [17].

Although there were many studies on properties of DRC, little data were available regarding the effect of using different nanoparticles (ZrO_2_, TiO_2_, and SiO_2_) with different concentrations on the properties of DRC. So, the current study aimed to assess the effect of 3 wt.% and 7 wt.% concentrations of ZrO_2_, TiO_2_, and SiO_2_ nanoparticles on the flexural strength (FS), hardness, and wear resistance of light cured DRCs. The null hypothesis of this study was that different nanoparticles (ZrO_2_, TiO_2_, or SiO_2_) with different concentrations (3 wt.% and 7 wt.%) would have no significant effect on FS, hardness, and wear resistance of the light cured DRCs.

## 2. Materials and Methods

Materials used in the current study, their types, chemical compositions, and manufacture's specifications are listed in Table 1. Composite specimens were fabricated in specific dimensions per test according to ISO and ADA specifications. For FS, rectangular specimens were prepared with specific dimensions (25 × 2 × 2 ± 0.03 mm) [18]; disc-shaped specimens were prepared for hardness and wear test with specific dimension (6×4 ± 0.03 mm) [19,20].

Sample size was determined by taking the necessary values from previous studies (*n* = 10, 70/test), so total sample size was two hundreds and ten specimens (210 specimens) [21, 22] .

All specimens were produced by the same investigator to reduce the variability, according to the nanoparticles type; samples were divided into four groups, 3 modified groups (ZrO_2_, TiO_2_, and SiO_2_) and one control group (without filler). Furthermore, each group was subdivided according to nanoparticles concentrations (3 wt.% and 7 wt.% nanoparticles). The filler volume fraction (vol%) of the inorganic filler was calculated as follows [23]:

(1) $$\begin{array}{c} Filler\;vol\%=\left(\frac{d_{r}\times wt\%}{d_{r}\times wt\%+d_{f}\left(100-wt\%\right)}\right)\times 100, \end{array}$$

where *d*_*r*_ is the density of the resin and *d*_*f*_ is the density of filler as shown in Table 2.

ZrO_2_, TiO_2_, and SiO_2_ nanoparticles were treated separately by using silane coupling agent [3-Trimethoxysilyl propyl methacrylate (TMSPM)] (Shanghai Richem International Co., Ltd., Shanghai, China) for creating reactive group to allow better adhesion between nanoparticles and resin matrix. TMSPM was dissolved in acetone to ensure that it would evenly coat the surfaces of nanoparticles which were added to the TMSPM/acetone solution and stirred with a magnetic stirrer (HS–350C, Yining, China) for 60 min; then the solvent was eliminated using a rotary evaporator (Rotavapor® R-300, Buchi AG, Flawil, Switzerland) under vacuum for 30 min at 60°C and 150 rpm. As the mixture was dried, it was heated at 120°C for 2 h and then bench-cooled to room temperature to get surface-treated nanoparticles [9,10]. The suitable amount of silane coupling agent (*X*) required for efficient and uniform coverage of nanoparticles was calculated by the following equation [9]:

(2) $$\begin{array}{c} X=\left(\frac{A}{\omega }\right)f, \end{array}$$

where *A* is the surface area of nanoparticles (m^2^/g), *ω* is the surface coverage per gram of silane (*ω* = 2525 m^2^/g), and ƒ is the amount of nanoparticles (g).

Suitable amount of silanated nanoparticles had been weighed by electronic balance of 0.001 gm accuracy (Denver instrument, Göttingen, Germany) to be incorporated in 3 wt.% and 7 wt.% concentrations of composite resin as mentioned in Table 3. Each treated nanoparticles powder was manually blended separately with composite resin material in glass beaker using a glass rod (7 mm diameter) for 5 min. at room temperature under day light until a homogeneous mix characterized with uniform color was obtained. A Teflon split mold was prepared with specific dimensions according to each test for packing the DCRs using Mylar strips [9,22].

Specimens were light cured from both sides for 20 sec. with light-curing unit (Mega-Physik dental, Rastatt, Germany), curing tip was positioned perpendicular to specimens' surfaces at zero distance to ensure complete polymerization of composite specimens, and then Mylar strips were removed and specimens were carefully extruded from their molds, excess material was removed using finishing discs (SofLex, 3M ESPE, Dental Products, St Paul, USA), and then polishing was done using silicon sand paper grit-600 and grit-800 (3M ESPE, USA) with a polishing machine (MetaServ 250 Grinder-Polisher, Buehler, Lake Bluff, USA) at 250 rpm in wet conditions and 30 sec for each step and discs were changed after three usages [24].

All specimens were visually inspected for any defects. Specimens with surface defects, broken edges, warpage, and porosities were excluded from the study. Passed specimens were digitally measured by a caliper (Mitutoyo Corp, Tokyo, Japan) with precision of 0.01 mm. Specimens were stored in distilled water (37°C for 48 h) according to ADA to simulate oral conditions [20].

Flexural test was applied by using three-point bending test with universal testing machine (Model LRX plus, Ametek instruments, Fareham, England). Each specimen was horizontally mounted in a custom-made loading fixture with the aid of a jig on a computer-controlled material testing machine with load cell of 5 KN. The load was set at zero and then increased gradually until the specimen failure at a crosshead speed of 5 mm/min. At the point of fracture, the maximum force (*N*) was recorded and flexural strength (FS) was calculated from the following formula [25]:

(3) $$\begin{array}{c} FS\left(\sigma \right)=\frac{3Fl}{2wh^{2}}, \end{array}$$

where *F* is the maximum load (*N*) excreted on specimen, *l* represented the distance (mm.) between two supports, *w* is the specimen width (mm.), and *h* is the specimen thickness (mm.).

For hardness test, specimens were further polished using silicon sand paper grit-1000 as mentioned by previous study to exclude the effect of surface roughness on the hardness values [26]. Microhardness Vickers Tester (Laizhou Huayin Testing Instrument Co., Ltd. Model Hvs-50, China) with diamond indenter and a 20X objective lens was used for hardness measurement. Five indentations with 200 gm of load for 10 sec were applied on the top surface of each specimen (the bottom surface was labeled) and placed at equal distance from each other and not closer than 0.5 mm to the adjacent ones or to specimen margin and then the average was calculated. The diagonal length of indentations was measured using built-in scaled microscope (Brunel Microscope Ltd., England) and the following equation was used to convert the obtained data into HV values [22]:

(4) $$\begin{array}{c} HV=1.854\left(\frac{P}{d^{2}}\right), \end{array}$$

where HV is Vickers microhardness in HVN = Kg/mm^2^, *P* is the load (Kg), and *d* is the length of the diagonals (mm.).

**Figure 1:**
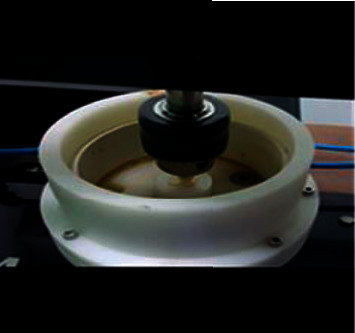
Upper antagonist and lower sample holder of chewing simulator.

For the wear test, 2-body wear test was achieved using a programmable logic-controlled equipment; 4-station multimodal ROBOTA chewing simulator integrated with thermocyclic protocol operated on servo-motor (Model Ach-09075Dc-t, AdTech Co. Ltd, Germany). Each compartment consists of upper Jacobs chuck as natural tooth antagonist holder that can be tightened with a screw and lower plastic sample holder in which specimen was embedded in a round Teflon housing by means of epoxy resin material (Technovit, Heraeus, Kulzer, Germany) (Figure 1). A weight of 700 gm. which is comparable to 7 N of chewing force was exerted and repeated 10000 times, which clinically simulates approximately one month of chewing function. In the present study, wear resistance was measured by evaluating surface roughness before and after wear simulation (Δ Ra); the parameters of wear test are listed in Table 4 [27,28].

**Figure 2:**
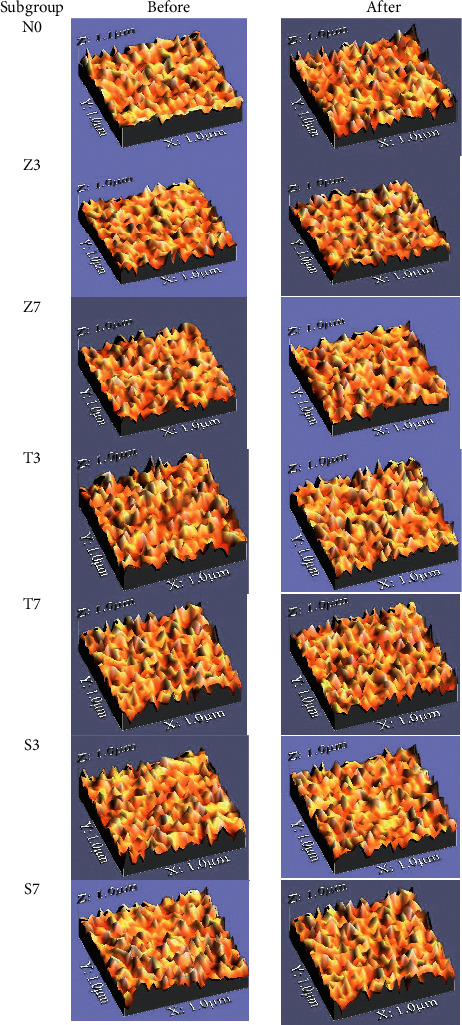
Digital image before and after wear simulation for different composite subgroups.

The sample was photographed before wear simulation using USB digital microscope with a built-in camera (Scope Capture Digital Microscope, Guangdong, China) connected with an IBM compatible personal computer (Dell, Inspiron15, China) using a fixed magnification of 120X. The image was recorded with a resolution of 1280 × 1024 pixels/image and then cropped to 350 × 400 pixels using Microsoft office picture manager (Microsoft corporation, 14.0.2015,SP2) to specify/standardize area of roughness measurement and then analyzed using WSxM software (Ver 5 develop 4.1, Nanotec, Electronica, SL.) in which all limits, sizes, frames, and measured parameters are expressed in pixels which are used to calculate average of heights (Ra1); subsequently, a 3D image of the surface profile of the sample was created using a digital image analysis system (Image J 1.43 U, National Institute of Health, USA) (Figure 2). 3D images were collected for each specimen and mean of surface roughness (*μ*m) was calculated by averaging three readings of each specimen (at center and both sides) [17,27,29].

After wear simulation, the testing device was stopped, specimens' surfaces were cleaned by a brush to remove any external particles or debris, and then each specimen was photographed again as previous to record (Ra2). The alteration in surface roughness records before and after wear replication was calculated according to the equation [30]:

(5) $$\begin{array}{c} \Delta Ra=Ra2-Ra1, \end{array}$$

where Ra is a unit of the arithmetical average of all departures of the profile through the mean sample length in *μ*m.

Data were presented as mean and standard deviation (SD) values. Statistical analysis was done using an analysis software program for Windows (SPSS Statistics, version 23; IBM Corp., USA). Data were checked for their normality distribution using Shapiro-Wilk and Kolmogorov-Smirnov tests. All data displayed parametric (normal) distribution. Two-way ANOVA was used to show the effect of different nanoparticle types (ZrO_2_, TiO_2_, and SiO_2_) and concentrations (0%, 3%, and 7%) on the different tested properties. Furthermore, one-way ANOVA was used to compare between all tested groups. Bonferroni's post hoc test was done for pairwise comparisons when ANOVA test is significant. The significance level was set at *p* ≤ 0.05.

## 3. Results

Mean, standard deviations (SD), and significant difference between groups for all tested properties were summarized in Table 5.

Two-way ANOVA test for FS showed that there was a significant interaction between type of nanoparticle and concentration of nanoparticles (*p* < 0.001) (Table 6). 1-way ANOVA showed statistically significant difference between FS of different groups (*p* < 0.001). In comparison with control group, all modified groups showed significant enhancement in FS except T7 and S7. Z3 and Z7 showed significant higher mean FS values than other subgroups; however T3 and S3 showed significant higher FS values than control group.

Two-way ANOVA test for the hardness showed that both type of nanoparticles and concentration of nanoparticles had a significant effect (*p* < 0.001) (Table 7), while the effect of their interaction was not statistically significant (*p*=0.142). 1-way ANOVA showed a statistically significant difference in hardness of different groups (*p* < 0.001). In comparison with control group, all modified groups showed statistically significant improvement in hardness where Z7 showed significantly higher mean hardness value than other subgroups; however Z3, T3, T7, S3, and S7 showed significantly higher hardness values than control group.

Two-way ANOVA test for wear resistance showed that there was a significant interaction between type of nanoparticles and concentration of nanoparticles (*p* < 0.001) (Table 8). 1-way ANOVA showed a statistically significant difference between ΔRa of different groups (*p* < 0.001). In comparison with control group, all modified groups showed significant enhancement in wear resistance except T7 and S7 where Z3, Z7, T3, and S3 which showed the statistically significant lowest mean ΔRa value (highest wear resistance); however T7 and S7 showed the lower mean ΔRa; digital images of T7 and S7 exhibited a relatively coarse and wrinkled surface pattern when compared with other groups (Figure 2).

## 4. Discussion

The composite resin is widely used in dentistry either as restorative material or as artificial teeth in the prosthodontics [2–5,31], and in the trial to improve its mechanical properties this study was carried out to appraise the effect of addition of 3 wt.% (0.78 vol%, 1.1 vol%, 2.27 vol%) and 7 wt.% (1.88 vol%, 2.58 vol%, 5.34 vol%) concentrations of ZrO_2_, TiO_2_, and SiO_2_ nanoparticles (respectively) on the FS and surface properties of light cured DRCs. Based on the results of our study, the addition of ZrO_2_, TiO_2_, and SiO_2_ nanoparticles significantly affected the flexural strength, hardness, and wear resistance of light cured DRCs; thus, the null hypothesis was rejected.

As nanoparticles were used for development of modified nanocomposite with improved physical and mechanical properties, it was established that the amount and dispersion of nanoparticles were the main reasons for improving the properties of composite resin, as decreasing the size and increase in the volume of fillers will lead to increase in surface hardness and compressive strength of the composite [32].

According to the most common concentrations investigated in the previous studies, two concentrations (3 wt.% and 7 wt.%) of nanoparticles were chosen also; it was found that percentage above 7 wt.% may cause massive change in the color of modified nanocomposite [21,33].

In the present study the FS of the specimens of control group was lower than that of ISO; also others reported that the FS of DRCs was lower than that of ISO [34,35].

The outcomes of current study showed a variable effect between different nanoparticles on the FS of DRCs; both concentrations of ZrO_2_ and 3 wt.% of TiO_2_ and SiO_2_ showed an increase in the FS of composite resin. The FS of all tested groups was higher than 80 MPa which was compatible with ISO reference [20].

The maximum FS value was observed in composite containing 7 wt.% ZrO_2_ indicating that the enhancement was concentration dependent; this may refer to uniform distribution of too small sized ZrO_2_ nanoparticles used in this study which allowed them to seal spaces between the linear chains of polymer matrix resulting in limiting the segmental motion of macromolecular chains which enhanced flexural strength [36].

Also, the increase in FS values may be due to transformation toughening of ZrO_2_; when sufficient stresses were developed and microcrack begun to propagate, ZrO_2_ nanoparticles transformed from tetragonal to monoclinic crystalline, depleting the energy of microcrack and arresting its propagation [37]. These findings were in agreement with many previous studies [38–40].

In contrast to the present findings, Rafid found that 1 and 3 wt.% of ZrO_2_ positively affect physical and mechanical properties of composite resin while high percentage (5, 7, or 10 wt.%) would adversely affect them [21]; the reason for this disagreement may be attributed to different methodology or different brands of composite material and nanoparticles.

The present study reported an increase in the FS with low 3 wt.% of Tio_2_ which come in agreement with Xia et al. and Hua et al. who found a significant increase in FS of composite resin reinforced with TiO_2_ nanoparticles and attributed that to the idea that TiO_2_ enables load relocation from resin matrix to NPs leading to improved mechanical properties of nanocomposites [13,41]. However, 7 wt.% of Tio_2_ reported a decline in FS which may be attributed to particle agglomeration [42].

The results of the present study showed that low concentration (3 wt.%) of SiO_2_ improved FS of composite resin as the filler dispersed properly in the resin matrix while, with high concentration (7wt.%), the system made more isolated particles and the curing power of composites decreased; thus FS of nanocomposite resins was reduced; this comes in line with many previous studies [34,43].

The findings of the present study showed an improvement in FS with both concentrations of ZrO_2_ while a declining occurred with high concentration of TiO_2_ and SiO_2_ which may be attributed to inherent properties and different particle size of each nanoparticle used [32,44].

On the other hand, the results of this study were at variance with other studies that found a significant increase in FS and hardness of composite modified with high concentrations of SiO_2_ (20–50 wt.% of a size ranging from 20 to 50 nm) [45,46]. Also in contrast to the results of the current study, others showed a negative effect on FS of composite resin reinforced with of SiO_2_. The reason for this disagreement is attributed to different methodology and different particle size [47].

Hardness of DRCs materials is a good indicator of their clinical performance as it indirectly reflects the extent of polymerization and predicts its wear resistance [48]. According to results of present study, ZrO_2_, TiO_2_, or SiO_2_ nanoparticles could increase the hardness of modified nanocomposite compared to unmodified specimens in a direct relation to concentrations. This finding was in accordance with many previous studies [16,48]. The obtained values of all tested specimens were more than 40 kg/mm^2^, at acceptable range provided by ISO [20].

The highest hardness value was obtained with 7 wt.% ZrO_2_ which could be explained by inherent properties of ZrO_2_ which considered the hardest nanoparticle among other metal oxides. In addition, this enhancement may be attributed to uniform dispersion of rigid inorganic nanoparticles and strong interfacial interactions between modified nanoparticles and resin matrix [44]; this was in accordance with previous studies [38,39].

The results of the present study showed an increase in hardness property with the addition of both concentrations of TiO_2_. Several studies revealed a significant rise in hardness and FS of composite resin modified with TiO_2_ [10,16]. Also, Xia et al. stated a significant increase in hardness of composite resin strengthened with 1 wt.% of TiO_2_ compared to unfilled composite [13].

The findings of the present study showed an increase in the hardness with the addition of both concentrations of SiO_2_. In agreement with these findings, Liu et al. studied the effect of different concentrations of SiO_2_ and found a significant increase in hardness of dental composite [16].

Two-body wear test was used in the present study during wear testing to simulate the two-body wear that normally occurs in the oral cavity during swallowing and parafunctional [49].

Rising wear resistance may add to the longevity of restorative materials through establishing durable esthetics and function. After clinical use, an inverse correlation was found between wear resistance and surface roughness of DRCs; increasing in wear resistance will lead to lowering roughness; Janus et al. proposed that usage of nanoparticles reduced surface roughness [50].

Based on the results of the current study, incorporation of both concentrations of ZrO_2_ and low concentration (3 wt.%) of TiO_2_ or SiO_2_ could improve the wear resistance of light cured composite resin; this may be explained by presence of harder nanoparticles on the specimens' surface which was difficult to exfoliate, retaining their surface integrity [51].

Meshref et al. stated that the reason for the decrease in wear of nanocomposite was attributed to increasing its hardness; this comes in agreement with the present findings regarding hardness and wear resistance [52]. Musanje and Ferracane found that incorporation of silanized nanoparticles significantly increased wear resistance of hybrid composite [53]. In addition, Manhart et al. found that wear resistance of composite resins was significantly enhanced with increased filler loading and decreased average filler particle size [54].

It was found that composite filled with 3 wt.% of ZrO_2_ exhibited the best wear resistance compared to unfilled one and the worn surface was smoother and flatter with very less voids [33].

In agreement with our results, others reported that addition of silanated TiO_2_ nanoparticles to dental composite improved its hardness and wear resistance and reduced polymerization shrinkage; this is attributed to better bonding of treated nanoparticles to resin matrix [13,52,55,56].

Guo et al. and Wang et al. proved that SiO_2_ was useful in improving the wear resistance of DRCs [57,58]. On the other hand, Han et al. concluded that nanofillers insignificantly affect the wear resistance of DRCs but may enhance the surface roughness of DRCs [59].

The differences in the results of our study and others [21,45–47] may be due to the differences in the materials used as well as methodology applied.

From the clinical point of view, improve the mechanical properties of the DRCs as fracture resistance, abrasion resistance, and hardness using small amounts of inorganic filler particles that remain the main target of the researchers to enable the DRCs to be used for both anterior and posterior restorations.

Using one type of DRC, lack of simulation of clinical and oral condition, and the thermocycling not performed were considered as the main limitations of this study, so clinical studies in different oral conditions are needed to support these in vitro results; also, further investigations using different types of composite materials are recommended. Caution was necessary when selecting the appropriate concentration and types of nanoparticles that will create a balance between achieved mechanical and optical properties of dental composite.

## 5. Conclusion

Concerning the results of the present study and according to its limitations, the following was concluded:

- The incorporation of 3 wt.% ZrO_2_, 7 wt.% ZrO_2_, 3 wt.% TiO_2_, and 3 wt.% SiO_2_ nanoparticles significantly increases the flexural strength and wear resistance of composite resin.
- The incorporation of 3 wt.% or 7 wt.% of ZrO_2_, TiO_2_, or SiO_2_ nanoparticles significantly increases the hardness of composite resin.
- 7 wt.% concentration of ZrO_2,_ may be beneficial in improving mechanical properties of composite resin while SiO_2_ and TiO_2_ are recommended in low concentrations (3 wt.%).

## Figures and Tables

**Table 1:** The study's materials.

Trade name	Manufacturer	Specifications
Nexcomp	META BIOMED, Korea	Resin: Bis-GMA, Bis-EMA, UDMA, TEGDMA
		Fillers: 0.04-0.7 *μ*m barium aluminum borosilicate, light cured A2 shade. Density 1.5 g/cm^3^
ZrO_2_ nanoparticles	NanoGATE, Cairo, Egypt	Spherical, white, and tetragonal particles with average size 12 ± 3 nm, density 5,89 g/cm^3^ at 25°C (purity >99%)
TiO_2_ nanoparticles	NanoGATE, Cairo, Egypt	Spherical, white, and anatase particles with average size 15 ± 3 nm, density 4,26 g/cm3 at 25°C (purity >99%)
SiO_2_ nanoparticles	NanoGATE, Cairo, Egypt	Spherical, white, and amorphous particles with average size 21 ± 3 nm, density 2 g/cm^3^ at 25°C (purity >99%)
Silane coupling agent	Sigma-Aldrich Chemie GmbH Riedstrasse 2, Germany	Purity 98%, ethanol 99.7%. lot no. 440159

**Table 2:** Grouping and coding of different variables.

	Group	Code	Description
No. 1	Control	N0	Unreinforced light cured composite resin
No. 2	ZrO_2_	Z3	Light cured composite resin reinforced with 3 wt.% (0.78 vol%) of ZrO_2_ NPs
		Z7	Light cured composite resin reinforced with 7 wt.% (1.88 vol%) of ZrO_2_ NPs
No. 3	TiO_2_	T3	Light cured composite resin reinforced with 3 wt.% (1.1 vol%) of TiO_2_ NPs
		T7	Light cured composite resin reinforced with 7 wt.% (2.58 vol%) of TiO_2_ NPs
No. 4	SiO_2_	S3	Light cured composite resin reinforced with 3 wt.% (2.27 vol%) of SiO_2_ NPs
		S7	Light cured composite resin reinforced with 7 wt.% (5.34 vol%) of SiO_2_ NPs

**Table 3:** Weight of nanoparticles and composite resin.

NPs concentration	Weight of composite (g)	Weight of NPs (g)
0 wt.% (control)	10	0
3 wt.%	10	0.3
7 wt.%	10	0.7

**Table 4:** Wear test's parameters.

Cold/hot bath temperature: 5°C/55^0^C	Dwell-time: 60 sec
Vertical-movement: 1 mm	Horizontal-movement: 3 mm
Rising-speed: 90 mm/s	Forward-speed: 90 mm/s
Descending-speed: 40 mm/s	Backward-speed: 40 mm/s
Cycle-frequency: 1.6 Hz	Weight/sample: 700 mg
Torque: 2.4 N.m	

**Table 5:** Descriptive statistics, one-way ANOVA, and pairwise comparisons for FS and surface properties of different subgroups.

Group	Flexural strength (MPa)	Hardness (VHN)	ΔRa (*μ*m)
	Mean ± SD		
N0	73.2 ± 8.5^D^	63 ± 1.8^C^	0.0028 ± 0.0004^C^
Z3	111.8 ± 8.5^B^	67.8 ± 1.9^B^	0.0019 ± 0.0002^A^
Z7	128.5 ± 8.9^A^	70.0 ± 2^A^	0.0022 ± 0.0005^AB^
T3	91.0 ± 2.9^C^	65.7 ± 1.6^B^	0.0018 ± 0.0003^A^
T7	80.7 ± 5.8^D^	66.3 ± 1.9^B^	0.0027 ± 0.0002^C^
S3	95.2 ± 2.8^C^	66.1 ± 1.3^B^	0.0020 ± 0.0003^AB^
S7	82.6 ± 3.3^D^	68.1 ± 0.9^B^	0.0026 ± 0.0004^C^
*P*-value	<0.001^*∗*^	<0.001^*∗*^	<0.001^*∗*^
*Effect size*	0.817	0.636	0.705

^*∗*^ *: Significant* at *P* ≤ 0.05, different superscripts vertically indicate statistically significant difference between groups.

**Table 6:** Two-way ANOVA test results for flexural strength (MPa).

*Parameter*	*Sum of squares*	*df*	*Mean square*	*f-value*	*p-value*
Type of nanofiller	14083.63	2	7041.82	90.8	**<0.001**
Concentration of nanoparticles	45.55	1	45.55	0.59	**0.447**
Type^*∗*^concertation	2579.26	2	1289.63	16.63	**<0.001**
Error	4187.71	54	77.55		

^*∗*^Significant (*P* ≤ 0.05).

**Table 7:** Two-way ANOVA test results for hardness (VHN).

*Parameter*	*Sum of squares*	*df*	*Mean square*	*f-value*	*p-value*
Type of nanoparticles	78.69	2	39.34	17.13	**<0.001** ^*∗*^
Concentration of nanoparticles	35.16	1	35.16	15.31	**<0.001** ^*∗*^
Type^*∗*^concentration	9.31	2	4.66	2.03	**0.142**
Error	124.05	54	2.3		

^*∗*^Significant (*P* ≤ 0.05).

**Table 8:** Two-way ANOVA test results for surface wear (*μ*m).

*Parameter*	*Sum of squares*	*df*	*Mean square*	*f-value*	*p-value*
Type of nanofiller	0.00	2	0.00	19.66	**<0.001** ^*∗*^
Concentration of nanoparticles	0.00	1	0.00	372.51	**<0.001** ^*∗*^
Type^*∗*^concertation	0.00	2	0.00	26.78	**<0.001** ^*∗*^
Error	0.00	54	0.00		

^*∗*^Significant (*P* ≤ 0.05).

## Data Availability

The data used to support the findings of the study can be obtained upon request from the corresponding author.
